# Targeting Lysyl Oxidase Family Meditated Matrix Cross-Linking as an Anti-Stromal Therapy in Solid Tumours

**DOI:** 10.3390/cancers13030491

**Published:** 2021-01-27

**Authors:** Yordanos F.I. Setargew, Kaitlin Wyllie, Rhiannon D. Grant, Jessica L. Chitty, Thomas R. Cox

**Affiliations:** 1 The Garvan Institute of Medical Research and The Kinghorn Cancer Centre, Darlinghurst, NSW 2010, Australia; y.setargew@garvan.org.au (Y.F.I.S.); k.wyllie@garvan.org.au (K.W.); rhiannon.grant@student.unsw.edu.au (R.D.G.); 2 St Vincent’s Clinical School, Faculty of Medicine, UNSW Sydney, Sydney, NSW 2052, Australia

**Keywords:** lysyl oxidases, extracellular matrix, cancer, tumour microenvironment, tumorigenesis, anti-stromal targeting

## Abstract

**Simple Summary:**

To improve efficacy of solid cancer treatment, efforts have shifted towards targeting both the cancer cells and the surrounding tumour tissue they grow in. The lysyl oxidase (LOX) family of enzymes underpin the fibrotic remodeling of the tumour microenvironment to promote both cancer growth, spread throughout the body and modulate response to therapies. This review examines how the lysyl oxidase family is involved in tumour development, how they can be targeted, and their potential as diagnostic and prognostic biomarkers in solid tumours.

**Abstract:**

The lysyl oxidase (LOX) family of enzymes are a major driver in the biogenesis of desmoplastic matrix at the primary tumour and secondary metastatic sites. With the increasing interest in and development of anti-stromal therapies aimed at improving clinical outcomes of cancer patients, the Lox family has emerged as a potentially powerful clinical target. This review examines how lysyl oxidase family dysregulation in solid cancers contributes to disease progression and poor patient outcomes, as well as an evaluation of the preclinical landscape of LOX family targeting therapeutics. We also discuss the suitability of the LOX family as a diagnostic and/or prognostic marker in solid tumours.

## 1. Introduction to the Matrix

The extracellular matrix (ECM) provides a supporting scaffold for cells to differentiate, communicate and grow within and plays an important function in maintaining tissue homeostasis [1,2]. This is achieved by a complex interplay between the fibrous proteins (such as collagens), glycoproteins, proteoglycans, and the bound organic (e.g., growth factors, cytokines) and inorganic (e.g., water, divalent cations) molecules of which the ECM is comprised [1]. Cells embedded within the ECM respond to biochemical and biomechanical properties of their surrounding environment and secrete a range of vital signaling molecules in order to communicate with neighboring cells [1,3,4]. Changes in the biomechanical and biochemical properties of the matrix influence key processes including proliferation and survival [5,6]. As a result of this multifaceted role in tissue homeostasis, dysregulation of the ECM can significantly disrupt normal tissue function and structure. Within the ECM, there is a range of molecules that provide both structural support as well as facilitating cell signaling. For example, collagen and glycoprotein/proteoglycan families engage cell surface receptors such as integrins and activate signaling networks, which consequently have multiple roles in modulating processes, such as further matrix assembly and remodeling, as well as cell behavior [7,8]. Fibrillar collagens and elastin largely make up the framework of the ECM that provides structural integrity and elasticity to tissue whilst simultaneously maintaining cell polarity and regulating migration [6]. Collagens, and in particular the fibrillar collagens, are important in conferring the biomechanical properties of the matrix. These mechano-activating properties in turn help to regulate, among other things, cell migration and proliferation [6,9].

Dysregulation of the deposition and composition of ECM components can both lead to and accelerate disease. For example, excessive deposition of collagens leads to fibrosis, characterized by a dense ECM. In solid cancers, the reprogramming of fibroblasts to cancer associated fibroblasts (CAFs) leads to desmoplasia, in which the ECM is remodeled by degradation of the existing, structured “normal” ECM and replaced by excessive, disordered “tumour” ECM that feeds into disease progression [10].

### 1.1. Collagen Biogenesis and Desmoplasia

**Figure 1:**
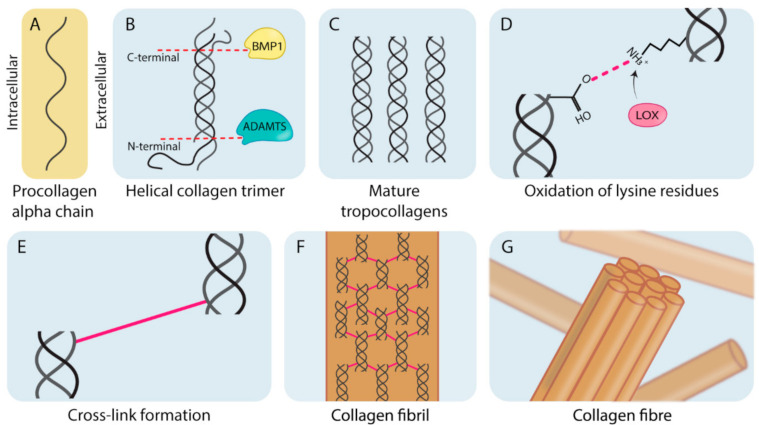
The biogenesis of fibrillar collagens. (**A**) Pro-collagen alpha-chain subunits are synthesized intracellularly by cells (predominantly fibroblast-like cells). (**B**) These alpha chains are assembled into helical collagen trimers in the endoplasmic reticulum and are then secreted into the extracellular space where they undergo post-translational processing by bone morphogenetic protein 1 (BMP1) and a disintegrin and metalloproteinase with thrombospondin motifs (ADAMTS). (**C**) Cleavage results in the formation of mature tropocollagen. (**D**) The lysyl oxidase (LOX) family members catalyze oxidative deamination of the lysine residues on the mature tropocollagen chains. (**E**) Deaminated residues on adjacent chains spontaneously condense and result in a cross-link. (**F**) Cross-linked tropocollagens organize to form collagen fibrils. (**G**) Collagen fibrils are further organized and assembled into collagen fibers.

Fibroblasts are the primary cell type involved in the production of matrix components such as fibrillar collagen. Collagens are typically heterotrimeric helical polymers (although some collagens form homotrimers) that form organized fibers comprised of bundled collagen fibrils (Figure 1) [3,10]. As shown in Figure 1, this process begins with synthesis of procollagen chains (Figure 1A). Collagens contain a glycine, proline (or hydroxyproline) repeating motif (Gly-Pro-X). Although this motif is conserved across all collagen types, variation in the organization and type of amino acid present in the X position in the different subunits allows for the generation of 28 molecularly different subtypes of collagen (Collagen I–XXVIII) [11,12]. Collagen subunits are formed from the single molecular chains by undergoing a number of processing steps. A critical initial step is the hydroxylation of proline and lysine residues in tropocollagen molecules (to form hydroxyproline and hydroxylysine, respectively), which is crucial in providing thermal stability to the final helical structure at biological temperatures required for healthy collagen formation [13]. Following from this, stable helical procollagen molecules are transported into the extracellular space where they are enzymatically cleaved at the C-terminal and the N-terminal domains by bone morphogenetic protein 1 (BMP1) and a disintegrin and metalloproteinase with thrombospondin motifs (ADAMTS) proteinases (Figure 1B). This produces mature tropocollagen molecules (Figure 1C) [14]. During fibril formation, the lysyl oxidase (LOX) family of enzymes catalyzes the formation of both intra and intermolecular cross-links which stabilize collagen molecules and fibrils, respectively [1]. This is achieved through LOX family oxidative deamination of lysine residues on tropocollagen chains (Figure 1D) which spontaneously form cross-links between tropocollagen molecules (Figure 1E), stabilizing them into collagen fibrils (Figure 1F). Collagen fibrils do not spontaneously assemble, and require the presence of, and binding to matrix glycoproteins to facilitate their assembly into parallel bundles that form mature collagen fibers (Figure 1G) [12,15].

### 1.2. The Lysyl Oxidase Family

One of the key enzymatically controlled stages of fibrillar collagen synthesis is the formation of stabilizing cross-links catalyzed by the lysyl oxidase (LOX) family of enzymes [16,17]. This LOX family consists of LOX and four LOX-like (LOXL) 1–4 isoforms. They are involved in one of the final post-translational modifications of collagen, where collagen fibrils, comprised of mature tropocollagen molecules bundled in parallel, are stabilized by the formation of covalent cross-links between adjacent fibrils (Figure 1) [9]. Similarly, the LOX family members also catalyze the oxidation of lysines in tropoelastin, resulting in stabilized cross-links between them to form strong, durable and flexible elastin microfibrils [16]. Effectively, these cross-links provide structural integrity to mature collagen and elastin sheets formed within the ECM, and may additionally protect these matrix molecules against proteolytic degradation [18,19].

Structurally, all LOX family members contain a highly conserved C-terminal copper binding domain consisting of three histidine residues which, when bound to copper, cause oxidation of Tyr355 and a conformational change of the active site to form a unique lysine tyrosylquinone (LTQ) cofactor. LOX and LOXL1 also both contain a cleavable, N-terminal pro-peptide domain cleaved by BMP1, whereas LOXL2-4 contain four repeating scavenger receptor cysteine-rich (SRCR) domains. In healthy tissue, the synthesis of the LOX family is tightly regulated to control the amount of active LOX family members present. The structure, genetic sequence and cross-linking activity of the LOX family has previously been extensively reviewed [16,20,21,22].

The degree of cross-linking of collagens and elastin within the ECM varies across tissue types and is thought to be related to the tensile strength required for tissue function [8,23]. LOX family expression and activity is regulated at both the transcriptional and post-translational levels (Section 2.1 and Section 2.2, respectively) [14,24,25,26,27,28]. It is for this reason that loss of regulation of LOX family mediated collagen cross-linking is known to be important in numerous pathologies such as tissue fibrosis and solid cancers. Herein, we give an overview of the role of the LOX family in the biogenesis of tumour matrix and its potential as a therapeutic target in cancer.

## 2. Dysregulation of the LOX Family in Solid Cancers

In cancer, dysregulation of the deposition, degradation, and post translational modification of collagens significantly alters the structure and function of the ECM [9,29]. As a key step in the regulation of collagen assembly, the LOX family has been reported to be dysregulated in a number of solid cancers. This is evidenced by excessive LOX family mediated cross-linking increasing stiffness of the ECM, and as a result promoting acquisition of malignant phenotypes and tumour progression in a number of tumour types [30,31]. Additionally, through cross-linking and subsequent modulation of ECM stiffness, the LOX family is involved in the modulation of cell proliferation and apoptosis, and loss of control of these pathways is considered a classical hallmark of cancer [32,33,34,35,36].

**Figure 2:**
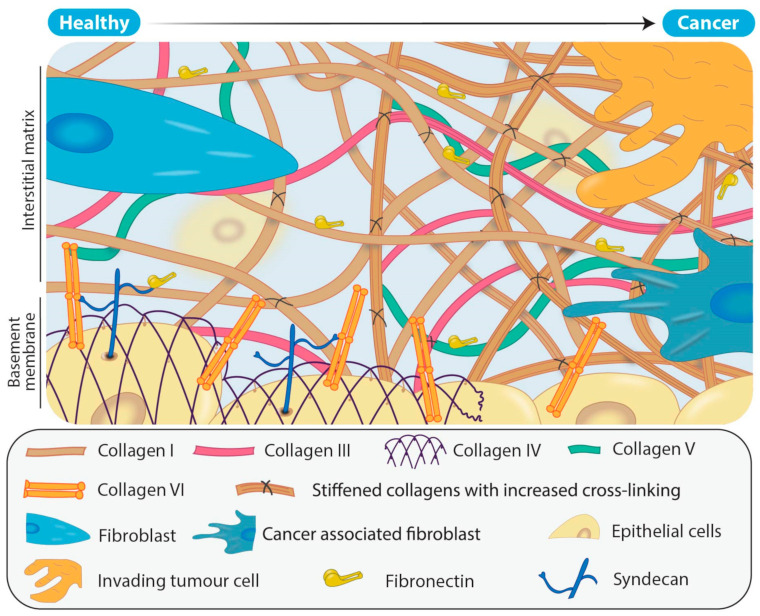
Remodeling of the extracellular matrix (ECM) in solid tumours. In healthy tissue, the ECM has a structured basement membrane consisting primarily of collagens IV and VI as well as a scaffolding arrangement of fibrillar collagens that are predominantly secreted by the fibroblasts. In comparison, solid tumours typically consist of more densely packed, aberrantly cross-linked fibrillar collagens resulting from the recruitment and activation of CAFs. As the level of deposition of fibrillar collagens such as collagens I, III and V increases in the tumour ECM, so too does LOX family mediated collagen cross-linking. In addition, tumour ECM results in a breakdown of the structure of the normal basement membrane.

Cancer associated fibroblasts (CAFs) are one of the most abundant types of stromal cell within the tumour microenvironment and are morphologically, epigenetically and metabolically distinct from their originating fibroblasts [37]. They promote the formation of a tumour supportive ECM through, among other actions, increased deposition of collagens, particularly type I, III and V fibrillar collagens (Figure 2) [38,39] and secretion of LOX family members, as well as chemokines and cytokines such as transforming growth factor-β (TGF-β), interleukin-6 (IL-6) and vascular endothelial growth factor A (VEGF) [29,40,41]. Simultaneously, CAFs secrete proteases (such as metzincins) responsible for degradation of the normal tissue ECM, and in particular collagens IV and VI that are major components of the basement membrane separating the epithelium from the underlying tissue (Figure 2). As a result, during tumour progression there is the gradual replacement of the originating tissue matrix with tumour matrix. This combined effect of increasing the deposition of fibrillar collagens and LOX family members, and degradation of key structural proteins, produces a disordered matrix and a lack of distinction between the basement membrane and now dense, disorganized interstitial matrix (Figure 2).

### 2.1. Interplay between Cell Signaling and LOX: Transcriptional Regulation

**Figure 3:**
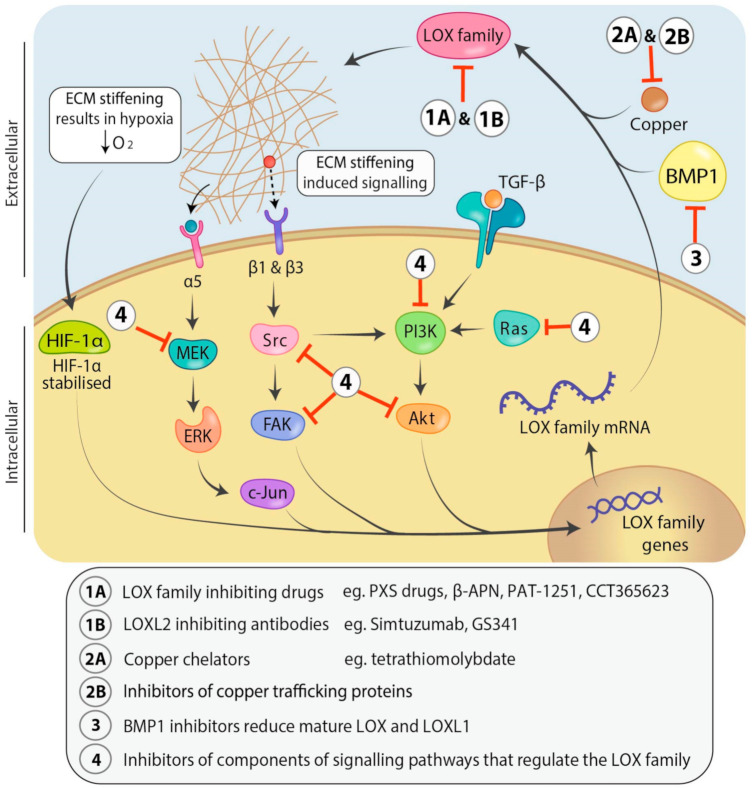
Signaling pathways involved in regulation of and therapeutic avenues for targeting the LOX family. Transforming growth factor-β (TGF-β) regulates LOX family expression through the phosphoinositide 3-kinase/protein kinase B (PI3K/Akt) signaling pathway. LOX family induced ECM stiffening causes mechano-activation of mitogen activated protein kinase (MAPK) and focal adhesion kinase/proto-oncogene tyrosine-protein kinase (FAK/Src) signaling pathways which are also known to upregulate LOX family expression, whilst decreasing oxygen diffusion into the tumour resulting in hypoxia. Hypoxia stabilizes hypoxia-inducible factor 1-alpha (HIF-1α) and leads to increased LOX and LOXL2 transcription through binding the hypoxia response elements in their promoters. LOX family members can be inhibited directly with small molecule drugs targeting their active sites (1A) or humanized antibodies that may bind directly or indirectly (allosterically) to the active sites of individual family members (1B). Alternately, LOX family members can be inhibited indirectly through use of copper chelators which remove the critical copper cofactor from the lysyl oxidase active site (2A), inhibitors of copper trafficking proteins (2B), inhibitors of bone morphogenetic protein 1 (BMP1) to prevent LOX and LOXL1 proenzyme processing and activation (3) or inhibitors of the components of the pathways involved in LOX family regulation (4). Abbreviations: Mitogen-activated protein kinase kinase (MEK), extracellular signal-regulated kinase (ERK), messenger RNA (mRNA).

LOX family members are upregulated in a number of different cancer types, and high levels of LOX family expression, as well as elevated deposition of fibrillar collagen in tumours, is associated with poor patient outcomes (see Table 1 and Table 2) [42,43,44,45]. Changes in LOX family member regulation, expression and subsequently enzymatic activity are therefore important factors in cancer progression. The tight regulation of LOX family expression (Figure 3) is controlled by a number of signaling pathways that, when dysregulated in cancer, can result in changes to expression of LOX family members. The hypoxic microenvironment found in solid tumours results in stabilization of hypoxia inducible factor 1α (HIF-1α) [25,46]. Both LOX and LOXL2 enzymes contain a hypoxia response element (HRE) in the promoter region of their genetic sequence which is important in transcriptional regulation [24,25,26]. When stable, HIF-1α binds to the HRE on the LOX and LOXL2 promoter [24,25,26], and directly regulates expression [25,26,47,48,49]. Interestingly, knockdown of LOX has been shown to downregulate HIF-1α expression [49,50], suggesting that LOX and HIF-1α may be involved in a positive feedback loop.

Potent fibrogenic signaling effectors such as TGF-β1 have been shown to be regulators of LOX expression in diseases such as cancer [79,80,81,82]. In addition, TGF-β1 has been shown to cause a dose-dependent increase in the expression levels and activity of LOX family members in a range of cell lines, including cardiac fibroblasts, liver cancer cells and hepatocellular carcinoma cells [24,79,80,81,82,83,84]. Upregulation of TGF-β in cancer is typically associated with activation of CAFs, increased deposition and stiffening of the ECM and subsequently tumour progression and metastasis [85,86]. TGF-β regulation of LOX family member expression appears to occur though PI3K/Akt signaling pathways downstream of the TGF-β receptor (Figure 3), with TGF-β causing an increase in Akt phosphorylation and subsequently LOX family expression in cardiac fibroblasts in 2D cultures [82,87], whilst inhibition of PI3K has been shown to decrease LOX expression in colorectal adenocarcinoma SW620 cells [82].

Expression of LOX family members typically causes stiffening of the ECM in cancer through increased catalytic activity, and this may result in further regulation of PI3K signaling though the mechano-activation of the FAK/Src pathway. Increases in LOX, LOXL2 and LOXL4 expression in the ECM of cancer is linked to an increase in focal adhesion kinase (FAK) and Src phosphorylation in colorectal adenocarcinoma [88,89], gastric cancer [53,56], clear cell renal cell carcinoma [90] and fibroblasts [91]. Furthermore, integrin β1 or β3 mediated mechanotransduction is correlated with LOX expression in clear cell renal cell carcinoma [90] and LOXL2 expression in fibroblasts [91], respectively, and is known to activate the FAK/Src or PI3K/Akt signaling pathways (Figure 3).

Additionally, LOX expression has been linked to MAPK signaling through p38-MAPK, ERK and JNK pathways in cardiac fibroblasts and breast cancer models [82,83]. In particular, increases in expression of c-Jun, a member of the JNK signaling pathway, corresponds with higher LOXL2 activity and appears to be activated by integrin α5 activation upon matrix stiffening in highly aggressive MHCC97H hepatocellular carcinoma cells [73]. In contrast, LOXL2 knockdown decreases integrin α5 activation of JNK signaling and results in reduced cell migration and focal adhesion formation in human clear cell renal cell carcinoma [90]. Overexpression of LOX in 4T1 breast cancer cells has been shown to increase activation of p38 MAPK signaling and was associated with greater cell invasion [83]. Inhibition of either p38-MAPK or ERK/2 has been shown to prevent TGF-β1 activation of LOX in cardiac fibroblasts [82], although whether this would also occur in a similar manner in CAFs in solid tumours remains to be seen. Collectively, these changes in signaling induced by LOX family expression and subsequent ECM stiffening typically serve to promote tumour progression.

### 2.2. Post-Translational Regulation of the LOX Family

LOX and LOXL1 are directly regulated at the post-translational level by BMP1 and ADAMTS proteinases, which are also responsible for cleaving collagen. Catalytically inactive LOX and LOXL1 are secreted as a pro-LOX into the extracellular space where BMP1 cleaves off the N-terminal pro-peptide, releasing the mature, active enzyme [27]. Cancer induced changes in the activity of these processing proteinases likely has a knock-on effect on LOX and LOXL1 collagen cross-linking activity. This effect has been shown in a model using mouse embryonic C3H10T1/2 cells which showed that increases in periostin, a protein which binds to BMP1 and enhances BMP1 deposition in the ECM. This subsequently results in more proteolytically cleaved, active LOX enzyme without an increase in LOX expression itself [92]. Effectively, this study showed that LOX activity and subsequent collagen cross-linking within the ECM could be altered through cancer induced changes to BMP1 production [92]. ADAMTS2 and 14 are also known to cleave the N-terminal domain of pro-LOX and, whilst BMP1 processing of LOX increases its binding affinity with triple helical collagen, further processing by ADAMTS2 and 14 reduces this binding [28]. It is unclear, however, what role ADAMTS processing may play in the activity and regulation of LOX and LOXL1 in solid cancers and more work is needed in this area. Overexpression of ADAMTS2 and 14 in gastric cancer [93] and breast carcinoma [94], respectively, was associated with poorer prognosis in patients, however, in oral squamous cell carcinoma, decreased expression of ADAMTS14 was associated with more advanced stage of disease and worse survival [95]. Although these studies did not directly examine changes to the LOX family, it is possible that dysregulation of ADAMTS2 and14 could be altering the regulation of LOX and LOXL1 and contributing to cancer progression reported and is an interesting area for further investigation.

## 3. Role of the Different LOX Family Members in Primary Tumour Development and Metastasis

**Figure 4:**
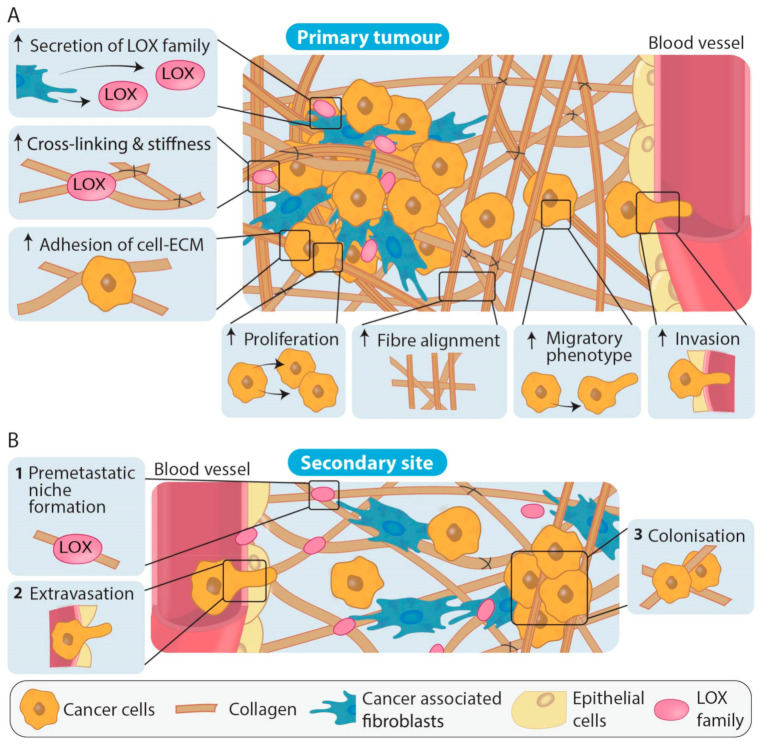
LOX family involvement in primary tumour formation and during the establishment of metastases. (**A**) Increased LOX cross-linking at the primary tumour site increases collagen fiber alignment as well as inducing multiple signaling cascades that result in the increased adhesion of cells to the ECM, increases in cancer cell proliferation, migration and invasion. Overall, LOX family actions result in changes in density and stiffness of the ECM that promote cancer cell dissemination to secondary sites. (**B**) At secondary sites, LOX family members are involved in generating a collagen rich ECM (1) and premetastatic niche formation through collagen remodeling (2). Extravasation of cancer cells to the premetastatic site and colonization of the cancer cells resulting in metastases (3) can then occur.

After embryonic development, LOX and LOXL1 can be found across various tissues including the heart, kidneys, testes, and uterus, whereas LOXL2, LOXL3 and LOXL4 are less widely expressed. Overall, LOX family activity is thought to be restricted until needed for processes such as maintaining tissue homeostasis and acute wound healing [96]. In tumour development and progression, LOX family expression and activity has largely been shown to be significantly increased (Table 1, Table 2 and Table 3). The various LOX family members have been identified as important players in stromal remodeling at the primary tumour which facilitates cancer progression in numerous cancer types, as summarized in Table 1, and therefore, these enzymes have potential as anti-stromal targets. Additionally, LOX family members secreted from primary tumour cells have been shown in in vivo models to contribute to reorganization of collagen and recruitment of CAFs and immune cells at secondary sites. The action of the LOX family members has also been shown to be important in premetastatic niche formation, an emerging concept wherein the ECM at future sites of metastatic colonization appears to be primed and remodeled prior to cancer cell colonization in order to optimize cancer cell survival and growth after extravasation [97,98] (Figure 4). These studies emphasize the importance of LOX family mediated ECM remodeling in cancer progression.

ECM remodeling fosters cancer growth and enhances cells’ abilities to metastasize [112,113,114,115]. However, complete ablation of the ECM does not simply cease tumour progression and improve survival outcomes [116,117]. An in vivo model selectively depleting myofibroblasts in pancreatic ductal adenocarcinoma (PDAC) showed that the corresponding decrease in collagen I did not improve the efficacy of gemcitabine chemotherapy. Rather, the depletion resulted in formation of a highly hypoxic, invasive and undifferentiated tumour leading to a reduced overall survival [117]. Similarly, another PDAC model which targeted the fibrotic stroma through sonic hedgehog ligand depletion showed that this depletion caused cells to favor other tumorigenic pathways also resulting in a more aggressive phenotype with poorer overall survival [118]. Therefore, more nuanced targeting of the ECM in cancer leading to stromal normalization rather than stromal ablation is likely required [119]. As a result, efforts have shifted towards identifying key targets involved in ECM remodeling in order to combine anti-stromal therapeutics with standard of care therapeutics to improve treatment outcomes [120,121,122].

## 4. Toward Using LOX Family Expression as a Diagnostic/Prognostic/Predictive Biomarker 

LOX family member involvement in shaping the ECM at both primary and secondary tumour sites (see Table 1), has resulted in an increased interest in determining their potential as companion biomarkers. The potential benefit of this is that early identification of aggressive malignancies is often critical in improving treatment outcomes and allowing clinicians to make informed treatment plans [123]. Additionally, tumour presentation varies across patients and differences in LOX family expression identified through current tissue biopsies holds potential to improve patient-based treatment plans, as patients could potentially be stratified based on their LOX family profile which may assist in determining suitability for anti-stromal therapies.

Since irregular LOX family expression has been linked to poor prognosis (Table 3), notably in breast, pancreatic, cervical, lung and liver cancer, as well as correlated with clinicopathological features of later stage disease in cervical, gastric and head and neck cancers [77,99,101,105,107,124,125,126,127,128], efforts towards using LOX family members for additional staging, as an indicator of disease progression, or a potential biomarker of response to therapy in solid cancers has increased. The relationship between LOX family member expression and activity on patient outcome and survival has been examined across numerous solid cancer types and is summarized in Table 3.

Given that current diagnostic and prognostic methods used in the clinic often require a tissue biopsy for histological analysis, the development of non-invasive or non-operative techniques would be favorable. LOX family members may have potential in this context since they are detectable in human bodily fluids such at the blood and urine. Some early preclinical success in this has demonstrated the detection of LOXL2 within both murine and human blood samples of breast cancer patients, where hydrogen peroxide release, a product of LOX family activity, was detected via the use of a gold-based electrochemical biosensor [129]. In this study, the biosensor was able to distinguish high LOXL2 in urine samples from patients with breast cancer compared to control samples. In addition to this, a recent study in gastric cancer showed a correlation between LOX concentrations present in the serum of patients and the corresponding tissue collected from these patients, which showed significantly increased LOX expression and later stage of disease [109]. Furthermore, through blood sample collection, a recent study in colorectal cancer patients discerned that in patients with liver metastases, tumour associated neutrophils were expressing high LOXL4 [62]. Although promising, further validation of target specificity in such techniques is still required, and yet to be explored is the disparity between expression of LOX family members and the amount of active remodeling of the ECM compared to that detectable in plasma. In addition, since the LOX family is involved in wound healing and tissue regeneration, serum/plasma levels may also be reflective of unrelated tissue fibrosis, or related tumour-induced co-morbidities, and so care will need to be taken in assigning direct associations. Regardless of this, developing a minimally invasive tool to detect LOX family member expression/activity in patients to determine those that are at high risk of progressing to metastatic disease and/or relapse has strong potential in improving treatment outcomes.

The complexities of correlating LOX family expression and tumour staging are typified in prostate cancer, where high LOX expression has been correlated with advanced tumour stage; however, low LOX expression was associated with decreased overall survival [128]. In other cancers such as breast and pancreatic, higher LOX expression correlates with promotion of metastasis [60,78,129,130,131], induction of chemoresistance [43,45] and poor survival [44,60,129]. Therefore, the use of LOX as a biomarker is likely to be cancer type specific. Some studies have also shown LOX family members may exert different influences on cancer progression within a single cancer type. An in vivo study of non-small cell lung carcinoma (NSCLC) showed that downregulation of LOXL2 accelerated disease progression and, when examining tumours from patients, higher stage disease was correlated with low LOXL2 expression [105]. However, it has also been reported that elevated LOX and LOXL2 increased metastasis formation and were significantly correlated with poor prognosis in NSCLC patients [126,132]. This suggests that further validation is required to fully understand causality between LOX family expression and patient outcomes before clinical use.

Indeed, different LOX family members have been reported to act as tumour promoters or suppressors depending on the cancer type (See Table 3). Additionally, the pro-peptide region of LOX, once cleaved from the mature LOX enzyme, has been shown to act as a tumour suppressor, whereas the mature enzyme is generally accepted to act as a tumour promoter. In particular, the LOX pro-peptide has been shown to decrease ERK and Akt signaling [133,134,135,136] by inhibiting FGF-2 signaling upstream of these pathways in pancreatic, breast and prostate cancer cells [133,135]. This is particularly significant in Ras driven cancer, where Ras activation causes FGF signaling leading to ERK and Akt phosphorylation. The LOX pro-peptide has been shown to reduce the invasive phenotype and anchorage independent growth of Ras transformed lung cancer [137], pancreatic cancer [137], breast cancer [138] and fibroblasts [139] on soft agar and reduce expression and binding of Ras stimulated NF-κB [137,138], whereas mature LOX had the opposite effect. In vivo, MIA PaCa-2 pancreatic cancer and NF639 breast cancer cells showed reduce tumour weight after injection into nude mice followed by injection of recombinant LOX pro-peptide [134], or when infected with a LOX pro-peptide over-expressing virus [136,138]. These results suggest a potential role for the LOX pro-peptide in anti-cancer therapy and future drug development [22].

## 5. Impact of the Lysyl Oxidase Family on Current Treatment Approaches

In addition to CAF mediated fibrosis and the collagen cross-linking activity of the LOX family, the chemotherapy induced wound healing response which often occurs at both primary and secondary sites typically results in an increase in matrix deposition. As a result, this exacerbated change in ECM deposition and stiffness may contribute to the development of acquired chemoresistance, likely due to changes in cellular signaling. Evidence for this is supported in 2D models, where overexpression of LOX had no effect on cancer cell sensitivity to doxorubicin, compared to 3D models where the cells in a collagen matrix showed reduced sensitivity to doxorubicin following LOX overexpression [23]. Increased LOX activity has been linked to the development of tumour chemoresistance [42,43], and reduced accumulation of doxorubicin in in vivo 4T1 breast adenocarcinoma tumour engraftments [42] and a reduction in the growth inhibitory effects of gemcitabine in Colo-357 colorectal cancer engraftments [43]. Blocking LOX activity was shown to increase drug penetration into tumours in these mouse models and improved homogeneity of drug distribution [42,43]. Importantly, this effect was shown to be likely due to altered diffusion through the ECM, not due to altered vasculature in tumours [42]. Combination therapy of gemcitabine or doxorubicin with LOX inhibition or knockdown improved survival and reduced tumour growth in chemo-resistant mouse models of pancreatic ductal and breast adenocarcinoma [42,44,45], and reduced metastasis compared to chemotherapy alone [42].

Surgery can result in an increase in LOX family expression as a result of a wound healing response at the site of surgery [140]. Plasma of colorectal cancer patients post-surgery was injected into mice and resulted in increased levels of lung metastasis compared to baseline serum, and this increase in metastasis was ablated when serum was depleted of LOX [140]. Furthermore, patients who receive radiation therapy in treatment of cancer commonly develop fibrosis at the site of treatment [141,142] and this fibrosis is associated with increased LOX activity [143]. LOX inhibition may therefore improve outcomes of patients undergoing surgery or receiving radiation therapy.

Many tumours display increased chemoresistance in the presence of hypoxia, and the expression of LOX family members in some chemo-resistant tumours is upregulated in response to hypoxia [23,45]. Hypoxic conditions stabilize HIF-1α, which in turn leads to upregulation of a number of genes that assists in chemoresistance, DNA repair and cell survival [144]. HIF-1α also promotes LOX transcription [24,25,26], and this upregulation contributes to the generation of a dense ECM through increasing cross-linking activity. Subsequently, this may reduce vasculature within the tumour and act as a barrier that reduces diffusion of oxygen into the tumour, as well as increasing mechano-signaling to promote tumour cells proliferation and increase metabolic demand. Ultimately, this combined effect further increases hypoxia (Figure 3) and the resulting hypoxic stress can activate DNA damage repair mechanisms [145] which make cells less vulnerable to nuclear targeting therapeutics including gemcitabine. Targeting the LOX family may therefore reduce chemoresistance in some tumours.

Combination treatment of chemo-resistant tumours with doxorubicin and inhibitors of LOX, or components of the pathways involved in LOX regulation such as FAK or Src, have also been shown to decrease tumour growth, suggesting that LOX and FAK/Src signalling play a role in chemoresistance [45]. It is possible that FAK phosphorylation downstream of LOX upregulation and cross-linking activity may increase through activation of cytokines involved in triggering DNA damage repair [146]. Taken together, these studies show that LOX family activity can have an impact on current chemotherapeutic and radiation treatments. Inhibition of LOX, and thus its effects on the ECM, is showing promise in improving patient outcomes when used in combinations with current therapies by reducing chemoresistance and increasing diffusion of chemotherapeutics into the tumours. With further study into this area, it is possible that administering lysyl oxidase inhibitors in clinical practice will benefit patients who have limited tolerance to chemotherapy, or who are not responding well to standard of care therapies.

## 6. Approaches to Target the Lysyl Oxidases Directly and Indirectly

### 6.1. Direct Approaches

The well characterized beta-aminopropionitrile (β-APN) is a non-specific, irreversible pan-LOX family inhibitor which covalently binds to the active site of LOX family enzymes [147]. It was the first widely used LOX family inhibitor and is commonly used in in vitro and in vivo studies. Originally shown to be tolerated in high doses in patients, it was later shown that long-term use was associated with prohibitive reactions including lathyritic effects on the bone due to its inhibitory action against a range of amine oxidases and is therefore no longer used in the clinic [148]. However, the base structure of β-APN has become pharmacologically important and used to develop a suite of lysyl oxidase specific compounds with different structural alterations to improve specificity [149,150]. Haloallylamine based drugs were developed by Pharmaxis Ltd. (Sydney, Australia), including PXS-5120A [151] and PXS-5153A [152], both of which are orally administered, covalently bind to and inhibit LOXL2 and 3 and have been shown to reduce collagen cross-linking and reduction of Ashcroft scores in liver and lung fibrosis [151,152]. PXS-S1A/C [67,153] and PXS-5382A, which inhibit LOXL2, have also been shown to delay tumour growth and reduce collagen accumulation in orthotopic models of breast cancer [153], and in orthotopic LY2 oral cancer models [67], respectively. The safety of the oral LOXL2 inhibitor PXS-5382A is currently being investigated in a phase 1 clinical trial in healthy adults (Clinical trial identifier: NCT04183517).

The highly specific small molecule inhibitor of LOXL2, PAT-1251, which is based on a benzylamine with 2-substituted pyridine-4-ylmethanamines, was developed by PharmAkea (San Diego, CA, USA). Although found to be well tolerated and successfully passing through a phase I clinical trial (Clinical trial identifier: NCT02852551), to date no phase II clinical trials have been completed with PAT-1251. Pre-clinical studies in vivo of the aminomethiophene based LOX inhibitor CCT365623 showed inhibition caused delayed tumour development and reduced lung metastasis in mouse breast cancer models [154,155,156]. Additionally, CCT365623 was shown to have good stability and specificity to LOX [154,155,156] but has yet to be tested in a clinical setting.

Another LOXL inhibitor to show early promise in cancer treatment was the anti-LOXL2 monoclonal mouse antibody AB0023, which was effective in reducing intratumoral collagen density in vivo in a murine model of pancreatic cancer [157]. Following from this, a humanized version of this antibody, simtuzumab, was developed. Simtuzumab allosterically binds to the SRCR domain four of LOXL2 [158] and showed early promise in animal studies where it was well tolerated and showed improved survival in mice in combination with paclitaxel compared to the vehicle or paclitaxel alone in a disseminated model of bone metastasis of MDA-MB-231 cells [159]. However, clinical trials of the drug demonstrated no improvement in progression-free survival of pancreatic or colorectal adenocarcinoma patients when simtuzumab was administered in combination with gemcitabine or folinic acid, fluorouracil and irinotecan (FOLFIRI), respectively, compared to the chemotherapeutic drug alone [160,161].

Other anti-LOXL2 antibodies have been developed in mice, such as GS341 [162] which caused reduced collagen cross-linking in liver fibrosis [163]; however, humanized versions of these antibodies have yet to be developed and as such it remains unclear if they, as with simtuzumab, would fail to demonstrate the same improvement in survival of humans as seen in animal models. It is therefore vitally important that appropriate disease models that are as most likely to be translatable in a clinical setting are chosen for LOX family inhibition studies. It is also important to consider the effect of genetic and epigenetic differences between cancer patients, and the possible ways in which cancer cells may compensate for a depletion in one LOX family member by upregulation of other family members or mechanisms involved in collagen cross-linking. Given that the LOX family is upregulated in a healthy wound healing response, future phase II clinical studies should closely monitor the potential effect that inhibitors of the LOX family could have on wound healing in patients following tumour resection or radiotherapy.

A number of studies to date have examined the use of LOX family inhibitors in combination with current chemotherapeutic treatments simultaneously. Given that lysyl oxidase inhibitors reduce the fibrotic ECM and desmoplasia in cancer, it is possible that initial treatment with lysyl oxidase inhibitors prior to administering chemotherapeutics will improve patient outcomes by priming the cancer microenvironment.

### 6.2. Indirect Approaches

Downregulation of LOX family expression or activity through indirect mechanisms may similarly be beneficial in targeting the LOX family, especially if approaches can be administered in combination with chemotherapeutics (Figure 3). BMP1 is involved in the post-translational control of LOX and LOXL1 activity through cleavage of the pro-peptide domain in order to release the mature protein. Therefore, inhibition of BMP1 could result in a reduction of active LOX and LOXL1 activity. There has been little research thus far examining the use of BMP1 inhibitors in combination with chemotherapeutics to specifically treat LOX/LOXL1 overexpressing cancers. This may be due, at least in part, to the difficulty in finding inhibitors of BMP1 that are selective, potent and cost effective [164], and the role that BMP1 plays in a range of other vital cellular processes including bone and cartilage development and repair [165]. Alternatively, increases in BMP1 may have tumour suppressive effects in Ras transformed cancers, where post-translational processing in cells highly expressing LOX and LOXL1 would increase levels of the tumour suppressive LOX pro-peptide.

LOX family activity may additionally be targeted through limiting the copper available to the cells. Copper is critically required for correct folding of LOX family enzymes, and the catalytic activity of their active sites. The copper chelator ammonium tetrathiomolybdate (ATTM) has been shown to reduce bleomycin-induced pulmonary fibrosis in mice by blocking LOX family activity [166]. Furthermore, ATTM was shown to lead to reduced LOX activity and collagen cross-linking [71]. ATTM is administered orally to chelate dietary copper. This is advantageous as it allows for easy cessation of the next dosage to impede any negative side effects that may arise during treatment. Chan and colleagues [71] reported that, although ATTM showed no significant effect on primary tumour progression, lung metastases in pre-clinical models of metastatic breast cancer were significantly reduced in the ATTM treatment groups. This may suggest that ATTM would be most valuable when administered prior to tumour resection to combat early LOX family mediated ECM remodeling of secondary sites, although more tailored preclinical studies are required to further examine this.

D-penicillamine (D-pen) is another orally administered copper chelator, initially suggested to be a LOXL2 inhibitor. Previous work showed in an in vivo breast cancer model that treatment with D-pen significantly reduced the number of lung metastases formed in mice compared to control groups [100]. Rather than being LOXL2 specific further investigation determined that like ATTM, D-pen broadly inhibits LOX family members [149,167]. Arguably a downside of such copper chelators is they only offer a non-specific method to target the LOX family which may complicate treatment due to the varied role of the individual members of the LOX family in tumorigenesis and tissue repair. 

Taking a different approach, Mohankumar et al. [168] developed “M” peptides designed to bind to the copper binding region of LOX through competitive inhibition without leading to a secondary conformational change in the protein, which was shown to render recombinant human LOX inactive. These peptides reduced activity of extracellular LOX from human umbilical vein endothelial cells (HuVEC) conditioned media, but in vitro and in vivo studies in cancer have yet to be performed. Furthermore, the use of inhibitors against copper trafficking proteins, such as antioxidant 1 copper chaperone (Atox1) and copper chaperone for superoxide dismutase (CCS), have been shown to decrease extracellular LOX activity in HuVEC in vitro [169]. Inhibitors of Atox1 and CCS have also shown promise in treating leukemia and lung, breast and head and neck cancers [170], although the involvement of the LOX family was not examined in this study. This approach may similarly be useful in combination with chemotherapeutics when treating LOX family overexpressing cancers.

LOX family member expression can also be modified by targeting components in the pathways leading to their expression (Figure 3). For example, Ras activation causes an increase in PI3K/Akt signaling which results in LOX upregulation [87]. Inhibition of Ras with the small molecule inhibitor Kobe0065 caused a reduction in the levels of mature LOX in 3D culture and reduced metastasis of SW620 cells to the lungs of mice after tail vein injection [87]. Similarly, the LOX pro-peptide inhibits Akt and ERK1/2 signaling in Ras transformed cancers [133,134,135,136] and reduces tumour volume in vivo [134,136,138]. TGF-β1 mediated LOX increase in fibroblasts and colorectal adenocarcinoma can be blocked by inhibitors of PI3K, p38-MAPK, JNK and ERK1/2, respectively [81,82,87]. A range of inhibitors for PI3K and Akt, as well as MEK in the MAPK signaling pathway, are being investigated in clinical trials for potential in treating a range of cancers [171,172], as are inhibitors for FAK [173] and Src [174]. However, these drugs have not been tested in relation to LOX family overexpressing cancers, nor have their effects on the LOX family been specifically evaluated to date, and so further work is required to understand the effect these inhibitors may have on LOX family expression and the subsequent role that their inhibition specifically plays on cancer progression in the context of these inhibitors. Unfortunately, the broad range of downstream effects elicited by targeting these key signaling networks likely means that LOX family specific effects will be difficult to dissect.

## 7. Conclusions

The remodeling of the extracellular matrix in cancer plays an important role in controlling the progression of disease and influences cell growth, motility and survival. As such, combining conventional chemotherapeutic targeting of cancer cells in solid tumours with a stromal targeting agent is likely to improve patient outcomes by disrupting pro-tumourigenic ECM remodeling. The LOX family of enzymes are favorable targets for anti-stromal therapeutics due to their importance in cancer development and progression when compared to healthy state ECM. Each LOX family member is critically involved in matrix deposition, and subsequently matrix stiffening, directly influencing the invasive and proliferative abilities of cancer cells, as well as the generation of pre-metastatic niches that support metastatic colonization. Additionally, highly LOX family expressing tumours have increased LOX family levels detectable in plasma [140], and thus holds the potential for less invasive techniques to be used for stratification of high LOX family expressing patients prior to administration of anti-stromal therapies. Beyond this, LOX family activity is known to contribute to chemotherapeutic resistance through reducing drug diffusion in tumours and activating a range of signaling cascades underlying tumour expansion and metastatic dissemination. Therefore, disrupting ECM remodeling by specifically targeting the LOX family may facilitate greater drug perfusion of standard of care treatments and reduce development of chemoresistance in tumours.

Although no inhibitors of the LOX family have currently been approved for routine clinical practice, preclinical and clinical trials underway are developing LOX family inhibitors which have to date shown high specificity and low toxicity. Novel classes of LOX family inhibitors have been designed to target either single, dual, or multiple LOX family members and these high degrees of specificity towards individual members of the LOX family offer significant promise. With this in mind, studies where LOX family inhibition appears to be correlated with poorer outcomes in early clinical trials should be further evaluated. Some LOX family members have been identified as tumour suppressive or tumour promoting in different cancer types and so investigating the individual LOX family member contributions to a specific cancer is essential to determine the most effective LOX family targeting combination. Additionally, further preclinical in vivo investigation would be valuable to examine when best to administer LOX family inhibitors in order to block tumour progression. Significant changes in the ECM of the tumour microenvironment during progression means that treatment regimens for early, late and advanced stage patients is likely to differ.

Recent work has resolved the partial crystal structure of LOXL2 [175]. Whilst this structure does not show the active site confirmation, it is a step closer to understanding LOXL2 and LOX family structure, which would allow for development of more specific inhibitors through in silico docking studies.

Alternately, indirect inhibition of LOX family members through the downregulation or inhibition of enzymes or signaling pathways required for LOX family expression may prove to be viable with further preclinical investigation, however such methods often lack target specificity. Ultimately, efforts continue towards the development of a well-tolerated method of targeting LOX family activity in cancer, and further investigation into the feasibility of LOX family targeting in a range of cancers and the interplay between different family members in this process will be required in the future, particularly given the sometimes contradictory roles different members of the LOX family can play in tumorigenicity.

With these barriers to anti-stromal targeting in mind, it is essential that treatment is complemented with a LOX family specific companion biomarker. Preclinical research has increased in utilizing LOX family members for the development of non-invasive methods of diagnosing and staging disease. Due to the secreted nature of the LOX family members, their detectable presence in the blood, and the well-established correlation between LOX family enzyme expression and prognosis in many solid tumours, this unique family offers promise as an inexpensive and non-invasive companion biomarker for highly stromal tumours. Although current non-invasive diagnostic measures remain in the preliminary stages of development, the LOX family holds huge potential as stromal targets in cancer.

## Figures and Tables

**Table 1:** Reported LOX family member involvement at the primary tumour site.

Cancer	LOX Family Member	Direction/Nature of Change	Action at Primary Tumour
Breast cancer	LOX	High expression	Matrix stiffening and increased focal adhesion formation. Increased migration and release of inflammatory cytokines. LOX inhibition reduced these effects [31,51,52].
	LOXL2	High expression	Matrix stiffening and increased downstream FAK/Src pathway signalling [53].
	LOXL4	High expression	Regulates tumour growth. Knockdown increased expression and density of collagen I and IV. Potential tumour suppressive role [54].
Bladder cancer	LOXL1	Low expression/silencing	Tumour suppressive role: LOXL1 over expression suppressed Ras activation and reduced ERK phosphorylation leading to reduced growth [55].
	LOXL4	Low expression/silencing	Overexpression suppresses Ras activation and partially reduces ERK phosphorylation leading to reduced colony formation, suggesting a role as a tumour suppressor gene [55].
Gastric cancer	LOXL3	High expression	Upregulation correlates to greater depth of invasion into surrounding tissue [56].
	LOXL4	High expression	Activates FAK/Src pathway, increasing tumour cell migration. Also stimulates increased cancer cell proliferation [56].
Liver cancer	LOXL2	High expression	Promoted vasculogenic mimicry, expression of SNAIL and vascular E-cadherin promoting tumour growth [57].
	LOXL4	High expression	A potentially novel regulator of p53, reduced tumour growth. Overexpression induced apoptosis [58].
Melanoma	LOX	High expression	Highly expressed in tumour endothelial cells. Inhibition reduced angiogenesis and metastasis [59].
	LOXL3	High expression	Activation of BRAF pathway, melanocyte transformation and aided melanocyte evasion from cell mediated death [33].
Colorectal cancer	LOX	High expression	Feedback loop with HIF-1α increased expression and phosphorylation of Akt, Src, and FAK, driving cell proliferation and epithelial-mesenchymal transition (EMT) [60].
	LOXL1	High expression	Tumour suppressive; Overexpression inhibits tumour growth, tumorigenesis and negatively regulates the hippo-YAP pathway. Knockdown increased migratory ability of tumour cells [61].
	LOXL4	High expression	Higher expression observed in highly desmoplastic regions of tumours [62].
Thyroid cancer	LOX	High expression	Interaction with mutated BRAF drives a more aggressive phenotype and increased risk of recurrence in patients [63].
Lung cancer	LOX	High expression	Promoted tumour growth and correlated increased matrix metalloproteinase (MMP)-2 and MMP-9 protein expression [64].
	LOXL1	High expression	Induced collagen reorganization and fiber alignment that promotes cancer cell invasion [65].
	LOXL2	High expression	Upregulation correlates with increased collagen density and fiber linearization [66].
Head and neck cancer	LOXL2	High expression	Expression triggers increased proliferation and downstream ERK1/2 activation [67].
Ovarian cancer	LOX	High expression	Hypoxia induced overexpression of LOX leads to down regulation of E-cadherin and invasive abilities of cells [25].
Urinary cancer	LOXL2	High expression	Hypoxia induced overexpression of LOX leads to down regulation of E-cadherin and invasive abilities of cells [25].
Pancreatic cancer	LOX	High expression	Increased fibrillar collagen deposition. Conversely, inhibition increased immune cell recruitment, vascularisation and enhanced efficacy of gemcitabine [44].
	LOXL1	High expression	Upregulation of transcripts found in human PDAC [43].
	LOXL2	High expression	Upregulation reduces chemotherapy delivery due to excess collagen inducing vasculature collapse [43].

Focal adhesion kinase/proto-oncogene tyrosine-protein kinase (FAK/Src), extracellular signal-regulated kinase (ERK), zinc finger protein SNAI1 (SNAIL), B-Raf proto-oncogene (BRAF), hypoxia-inducible factor 1-alpha (HIF-1α), protein kinase B (Akt), yes associated protein (YAP), matrix metalloproteinase-2 and 9 (MMP-2, 9).

**Table 2:** Reported LOX family member involvement at the metastatic site.

Cancer	Lysyl Oxidase Family Member	Direction/Nature of Change	Action at Metastatic Site
Colorectal cancer	LOX	High expression	Tumour secreted LOX induced the production of IL-6 and activation of STAT, thereby promoting bone resorption, priming bone marrow for tumour cell colonisation [60].
	LOXL1	Low expression	Tumour suppressive; significantly downregulated in liver metastases and overexpression in vitro reduced migration and invasion of cells and in vivo suppressed metastasis [61].
	LOXL2	High expression	Over-expression upregulated vimentin and downregulated E-cadherin, thus increasing migratory potential of cells to favour metastasis [68].
	LOXL4	High expression	Neutrophils recruited to premetastatic niche increased expression of LOXL4 at metastatic sites [62].
Breast cancer	LOX	High expression	Induced TWIST1 expression promoting EMT [69]. Increased cross-linking of collagen in lungs, recruited CD11b+ cells, and triggered premetastatic niche formation. Silencing/inhibition reduced both tissue remodelling and metastasis formation [70,71].
	LOXL2	High expression	Induced higher VEGF expression in CAFs, promoting lymphangiogenesis and lymph node metastasis. Inhibition significantly reduced lung metastases [72].
Melanoma	LOXL3	High expression	Interaction with SNAIL downregulates E-cadherin and promotes EMT [8].
Liver cancer	LOXL2	High expression	Induced collagen remodelling and increased expression of MMP-9, stromal cell derived factor-1 and production of fibronectin at lungs, fostering metastatic colonisation [24,73].
Gastric cancer	LOX	High expression	Repression of E-cadherin promoting EMT. Inhibition significantly reduces migration of cells [74]. Induces Warburg effect through regulation of HIF-1α and c-Myc [75]. Niche formation: involved in degradation of collagen IV, MMP-9 and infiltration of macrophages [76].
	LOXL4	High expression	Interaction with FAK/Src pathway aided gastric cancer cell adhesion with fibronectin during metastasis [56].
Lung cancer	LOX	High expression	Induced EMT in cells promoting invasion/metastasis. Knockdown reduced migration of cells [66].
	LOXL2	High expression	Induced EMT. Knockdown reduced invasion of cells and metastasis in vivo. in vitro colonies formed are smaller in size compared to control [66].
Head and neck cancers	LOXL2	High expression	Interaction with SNAIL which downregulated E-cadherin, promoting EMT [67].
Cervical cancer	LOXL2	High expression	Correlated with promotion of proliferation and EMT in cells [77].
Pancreatic cancer	LOX	High expression	LOX knockdown reduced cancer cells ability to invade, and is correlated with reduced Src phosphorylation [44].
	LOXL2	High expression	Regulator of EMT, where inhibition significantly decreased cell proliferation, migration and invasion [78].

Interleukin-6 (IL-6), signal transducer and activator of transcription (STAT), Twist-related rptoein 1 (TWIST-1), epithelial-mesenchymal transition (EMT), vascular endothelial growth factor (VEGF), zinc finger protein SNAI1 (SNAIL), hypoxia-inducible factor 1-alpha (HIF-1α), matrix metalloproteinase-9 (MMP-9).

**Table 3:** LOX family members identified as a prognostic marker/biomarker of disease.

Cancer	LOX Family Member	Direction of Change	Outcome
Cervical cancer	LOXL2	High expression	Poorer overall survival, with more advanced tumours showing high LOX expression [99].
Breast cancer	LOX	High expression	Significantly associated with worse disease-free survival in chemotherapy resistant TNBC patients [45].
	LOXL1	High expression	Associated with high expression of fibrillar collagen and chemoresistance [42].
	LOXL2	High expression	Poor survival and increased metastases, and associated with chemoresistance [42,100].
	LOXL4	High expression	Attributed to significantly reduced survival [56].
Colorectal cancer	LOX	High expression	Associated with poor overall and disease free survival [60].
	LOXL1	Low expression	Significantly down regulated in patients with metastases [61].
	LOXL2	High expression	Associated with poorer overall survival, higher expression correlated with greater number of metastases [68].
Head and neck cancer	LOX	High expression	Shorter overall survival, significantly correlated with lymph node metastasis [101,102].
	LOXL3	High expression	Significant association with worse survival and higher risk of metastasis [103,104].
Non-small cell lung cancer	LOXL2	Downregulation/loss of function	Poorer pathological stage and differentiation [105].
Ovarian cancer	LOX	High expression	Associated with chemoresistance [42], poor overall and progression free survival in stage III/IV patients [106].
	LOXL1	High expression	Correlated with poor overall and progression free survival in stage III/IV patients [106].
	LOXL2	High expression	Correlated with poor overall survival in stage III/IV patients [106].
	LOXL3	High expression	Associated with poor overall and poor progression free survival in grade II/III and stage I/II patients [106].
Liver cancer	LOXL2 (intracellular)	High expression	Poorer prognosis, shorter overall survival, positively correlated with greater fibrosis and risk of recurrence [107,108].
	LOXL 4	Low expression	Reduced overall survival in patients [58].
Gastric cancer	LOX	High expression	Aggressive liver metastasis and reduced overall survival [75]. Expression level correlated with clinicopathological features of disease [76,109].
	LOXL4	High expression	Poorer overall survival, more advanced stage of disease and greater depth of tumour invasion [56].
Pancreatic cancer	LOX	High expression	Patients with high LOX profile have worse overall survival, disease free survival and greater metastatic burden [110].
	LOXL 2	High expression	Correlated with clinicopathological features of advanced disease and worse overall survival [78].
Urinary cancers	LOX	High expression	Correlated with poorer disease specific survival and progression free survival [111].
	LOXL2	High expression	Correlated with poorer survival at all stages of disease [111].

## Data Availability

No new data were created or analyzed in this study. Data sharing is not applicable to this article.

## References

[1] Theocharis A.D., Skandalis S.S., Gialeli C., Karamanos N.K. (2016). Extracellular matrix structure. Adv. Drug Deliv. Rev..

[2] Desmoulière A., Darby I., Costa A.M., Raccurt M., Tuchweber B., Sommer P., Gabbiani G. (1997). Extracellular matrix deposition, lysyl oxidase expression, and myofibroblastic differentiation during the initial stages of cholestatic fibrosis in the rat. Lab. Investig..

[3] Yamauchi M., Barker T.H., Gibbons D.L., Kurie J.M. (2018). The fibrotic tumor stroma. J. Clin. Investig..

[4] Ruprecht V., Monzo P., Ravasio A., Yue Z., Makhija E., Strale P.O., Gauthier N., Shivashankar G.V., Studer V., Albiges-Rizo C. (2017). How cells respond to environmental cues—insights from bio-functionalized substrates. J. Cell Sci..

[5] Pizzo A.M., Kokini K., Vaughn L.C., Waisner B.Z., Voytik-Harbin S.L. (2005). Extracellular matrix (ECM) microstructural composition regulates local cell-ECM biomechanics and fundamental fibroblast behavior: A multidimensional perspective. J. Appl. Physiol..

[6] Frantz C., Stewart K.M., Weaver V.M. (2010). The extracellular matrix at a glance. J. Cell Sci..

[7] Woods A. (2001). Syndecans: Transmembrane modulators of adhesion and matrix assembly. J. Clin. Investig..

[8] Amendola P.G., Reuten R., Erler J.T. (2019). Interplay between LOX enzymes and integrins in the tumor microenvironment. Cancers.

[9] Sherman V.R., Yang W., Meyers M.A. (2015). The materials science of collagen. J. Mech. Behav. Biomed. Mater..

[10] Komohara Y., Takeya M. (2017). CAFs and TAMs: Maestros of the tumour microenvironment. J. Pathol..

[11] Ricard-Blum S. (2011). The collagen family. Cold Spring Harb. Perspect. Biol..

[12] Collagen: The Fibrous Proteins of the Matrix—Molecular Cell Biology—NCBI Bookshelf. https://www.ncbi.nlm.nih.gov/books/NBK21582.

[13] Rappu P., Salo A.M., Myllyharju J., Heino J. (2019). Role of prolyl hydroxylation in the molecular interactions of collagens. Essays Biochem..

[14] Hopkins D.R., Keles S., Greenspan D.S. (2007). The bone morphogenetic protein 1/Tolloid-like metalloproteinases. Matrix Biol..

[15] Bi Y., Patra P., Faezipour M. (2014). Structure of collagen-glycosaminoglycan matrix and the influence to its integrity and stability. Annu. Int. Conf. IEEE Eng. Med. Biol. Soc..

[16] Chitty J.L., Setargew Y.F.I., Cox T.R. (2019). Targeting the lysyl oxidases in tumour desmoplasia. Biochem. Soc. Trans..

[17] Yamauchi M., Taga Y., Hattori S., Shiiba M., Terajima M. (2018). Analysis of collagen and elastin cross-links. Methods Cell Biol..

[18] Saini K., Cho S., Dooling L.J., Discher D.E. (2020). Tension in fibrils suppresses their enzymatic degradation—A molecular mechanism for “use it or lose it”. Matrix Biol..

[19] Schmelzer C.E.H., Heinz A., Troilo H., Lockhart-Cairns M.P., Jowitt T.A., Marchand M.F., Bidault L., Bignon M., Hedtke T., Barret A. (2019). Lysyl oxidase-like 2 (LOXL2)-mediated cross-linking of tropoelastin. FASEB J..

[20] Johnston K.A., Lopez K.M. (2018). Lysyl oxidase in cancer inhibition and metastasis. Cancer Lett..

[21] Vallet S.D., Ricard-Blum S. (2019). Lysyl oxidases: From enzyme activity to extracellular matrix cross-links. Essays Biochem..

[22] Trackman P.C. (2018). Functional importance of lysyl oxidase family propeptide regions. J. Cell Commun. Signal..

[23] Schütze F., Röhrig F., Vorlová S., Gätzner S., Kuhn A., Ergün S., Henke E. (2015). Inhibition of lysyl oxidases improves drug diffusion and increases efficacy of cytotoxic treatment in 3D tumor models. Sci. Rep..

[24] Wong C.C.-L., Tse A.P.-W., Huang Y.-P., Zhu Y.-T., Chiu D.K.-C., Lai R.K.-H., Au S.L.-K., Kai A.K.-L., Lee J.M.-F., Wei L.L. (2014). Lysyl oxidase-like 2 is critical to tumor microenvironment and metastatic niche formation in hepatocellular carcinoma. Hepatology.

[25] Schietke R., Warnecke C., Wacker I., Schödel J., Mole D.R., Campean V., Amann K., Goppelt-Struebe M., Behrens J., Eckardt K.-U. (2010). The lysyl oxidases LOX and LOXL2 are necessary and sufficient to repress E-cadherin in hypoxia: Insights into cellular transformation processes mediated by HIF-. J. Biol. Chem..

[26] Wang V., Davis D.A., Yarchoan R. (2017). Identification of functional hypoxia inducible factor response elements in the human lysyl oxidase gene promoter. Biochem. Biophys. Res. Commun..

[27] Uzel M.I., Scott I.C., Babakhanlou-Chase H., Palamakumbura A.H., Pappano W.N., Hong H.H., Greenspan D.S., Trackman P.C. (2001). Multiple bone morphogenetic protein 1-related mammalian metalloproteinases process pro-lysyl oxidase at the correct physiological site and control lysyl oxidase activation in mouse embryo fibroblast cultures. J. Biol. Chem..

[28] Rosell-García T., Paradela A., Bravo G., Dupont L., Bekhouche M., Colige A., Rodriguez-Pascual F. (2019). Differential cleavage of lysyl oxidase by the metalloproteinases BMP1 and ADAMTS2/14 regulates collagen binding through a tyrosine sulfate domain. J. Biol. Chem..

[29] Liu T., Zhou L., Li D., Andl T., Zhang Y. (2019). Cancer-Associated fibroblasts build and secure the tumor microenvironment. Front. Cell Dev. Biol..

[30] Chaudhuri O., Koshy S.T., Branco da Cunha C., Shin J.-W., Verbeke C.S., Allison K.H., Mooney D.J. (2014). Extracellular matrix stiffness and composition jointly regulate the induction of malignant phenotypes in mammary epithelium. Nat. Mater..

[31] Levental K.R., Yu H., Kass L., Lakins J.N., Egeblad M., Erler J.T., Fong S.F.T., Csiszar K., Giaccia A., Weninger W. (2009). Matrix crosslinking forces tumor progression by enhancing integrin signaling. Cell.

[32] Huang Z.M., Du S.H., Huang L.G., Li J.H., Xiao L., Tong P. (2016). Leptin promotes apoptosis and inhibits autophagy of chondrocytes through upregulating lysyl oxidase-like 3 during osteoarthritis pathogenesis. Osteoarthr. Cartil..

[33] Santamaría P.G., Floristán A., Fontanals-Cirera B., Vázquez-Naharro A., Santos V., Morales S., Yuste L., Peinado H., García-Gómez A., Portillo F. (2018). Lysyl oxidase-like 3 is required for melanoma cell survival by maintaining genomic stability. Cell Death Differ..

[34] Xu Y., Wang X., Huang Y., Ma Y., Jin X., Wang H., Wang J. (2018). Inhibition of lysyl oxidase expression by dextran sulfate affects invasion and migration of gastric cancer cells. Int. J. Mol. Med..

[35] Kim D., Mecham R.P., Trackman P.C., Roy S. (2017). Downregulation of lysyl oxidase protects retinal endothelial cells from high glucose-induced apoptosis. Investig. Ophthalmol. Vis. Sci..

[36] Kim B.-R., Dong S.M., Seo S.H., Lee J.-H., Lee J.M., Lee S.-H., Rho S.B. (2014). Lysyl oxidase-like 2 (LOXL2) controls tumor-associated cell proliferation through the interaction with MARCKSL1. Cell Signal..

[37] Bu L., Baba H., Yoshida N., Miyake K., Yasuda T., Uchihara T., Tan P., Ishimoto T. (2019). Biological heterogeneity and versatility of cancer-associated fibroblasts in the tumor microenvironment. Oncogene.

[38] Nissen N.I., Karsdal M., Willumsen N. (2019). Collagens and cancer associated fibroblasts in the reactive stroma and its relation to cancer biology. J. Exp. Clin. Cancer Res..

[39] Xu S., Xu H., Wang W., Li S., Li H., Li T., Zhang W., Yu X., Liu L. (2019). The role of collagen in cancer: From bench to bedside. J. Transl. Med..

[40] Vennin C., Mélénec P., Rouet R., Nobis M., Cazet A.S., Murphy K.J., Herrmann D., Reed D.A., Lucas M.C., Warren S.C. (2019). CAF hierarchy driven by pancreatic cancer cell p53-status creates a pro-metastatic and chemoresistant environment via perlecan. Nat. Commun..

[41] Liu T., Han C., Wang S., Fang P., Ma Z., Xu L., Yin R. (2019). Cancer-associated fibroblasts: An emerging target of anti-cancer immunotherapy. J. Hematol. Oncol..

[42] Rossow L., Veitl S., Vorlová S., Wax J.K., Kuhn A.E., Maltzahn V., Upcin B., Karl F., Hoffmann H., Gätzner S. (2018). LOX-catalyzed collagen stabilization is a proximal cause for intrinsic resistance to chemotherapy. Oncogene.

[43] Le Calvé B., Griveau A., Vindrieux D., Maréchal R., Wiel C., Svrcek M., Gout J., Azzi L., Payen L., Cros J. (2016). Lysyl oxidase family activity promotes resistance of pancreatic ductal adenocarcinoma to chemotherapy by limiting the intratumoral anticancer drug distribution. Oncotarget.

[44] Miller B.W., Morton J.P., Pinese M., Saturno G., Jamieson N.B., McGhee E., Timpson P., Leach J., McGarry L., Shanks E. (2015). Targeting the LOX/hypoxia axis reverses many of the features that make pancreatic cancer deadly: Inhibition of LOX abrogates metastasis and enhances drug efficacy. EMBO Mol. Med..

[45] Saatci O., Kaymak A., Raza U., Ersan P.G., Akbulut O., Banister C.E., Sikirzhytski V., Tokat U.M., Aykut G., Ansari S.A. (2020). Targeting lysyl oxidase (LOX) overcomes chemotherapy resistance in triple negative breast cancer. Nat. Commun..

[46] Choi Y.K., Kim C.-K., Lee H., Jeoung D., Ha K.-S., Kwon Y.-G., Kim K.-W., Kim Y.-M. (2010). Carbon monoxide promotes VEGF expression by increasing HIF-1alpha protein level via two distinct mechanisms, translational activation and stabilization of HIF-1alpha protein. J. Biol. Chem..

[47] Gao Y., Xiao Q., Ma H., Li L., Liu J., Feng Y., Fang Z., Wu J., Han X., Zhang J. (2010). LKB1 inhibits lung cancer progression through lysyl oxidase and extracellular matrix remodeling. Proc. Natl. Acad. Sci. USA.

[48] Pez F., Dayan F., Durivault J., Kaniewski B., Aimond G., Le Provost G.S., Deux B., Clézardin P., Sommer P., Pouysségur J. (2011). The HIF-1-inducible lysyl oxidase activates HIF-1 via the Akt pathway in a positive regulation loop and synergizes with HIF-1 in promoting tumor cell growth. Cancer Res..

[49] Ji F., Wang Y., Qiu L., Li S., Zhu J., Liang Z., Wan Y., Di W. (2013). Hypoxia inducible factor 1α-mediated LOX expression correlates with migration and invasion in epithelial ovarian cancer. Int. J. Oncol..

[50] Di Stefano V., Torsello B., Bianchi C., Cifola I., Mangano E., Bovo G., Cassina V., De Marco S., Corti R., Meregalli C. (2016). Major action of endogenous lysyl oxidase in clear cell renal cell carcinoma progression and collagen stiffness revealed by primary cell cultures. Am. J. Pathol..

[51] Salvador F., Martin A., López-Menéndez C., Moreno-Bueno G., Santos V., Vázquez-Naharro A., Santamaria P.G., Morales S., Dubus P.R., Muinelo-Romay L. (2017). Lysyl oxidase-like protein LOXL2 promotes lung metastasis of breast cancer. Cancer Res..

[52] Jeong Y.J., Park S.H., Mun S.H., Kwak S.G., Lee S.-J., Oh H.K. (2018). Association between lysyl oxidase and fibrotic focus in relation with inflammation in breast cancer. Oncol. Lett..

[53] Peng L., Ran Y.-L., Hu H., Yu L., Liu Q., Zhou Z., Sun Y.-M., Sun L.-C., Pan J., Sun L.-X. (2009). Secreted LOXL2 is a novel therapeutic target that promotes gastric cancer metastasis via the Src/FAK pathway. Carcinogenesis.

[54] Choi S.K., Kim H.S., Jin T., Moon W.K. (2017). LOXL4 knockdown enhances tumor growth and lung metastasis through collagen-dependent extracellular matrix changes in triple-negative breast cancer. Oncotarget.

[55] Wu G., Guo Z., Chang X., Kim M.S., Nagpal J.K., Liu J., Maki J.M., Kivirikko K.I., Ethier S.P., Trink B. (2007). LOXL1 and LOXL4 are epigenetically silenced and can inhibit ras/extracellular signal-regulated kinase signaling pathway in human bladder cancer. Cancer Res..

[56] Li R., Zhao W., Fang F., Zhuang C., Zhang X., Yang X., Jiang S., Kong F., Tu L., Zhang W. (2015). Lysyl oxidase-like 4 (LOXL4) promotes proliferation and metastasis of gastric cancer via FAK/Src pathway. J. Cancer Res. Clin. Oncol..

[57] Shao B., Zhao X., Liu T., Zhang Y., Sun R., Dong X., Liu F., Zhao N., Zhang D., Wu L. (2019). LOXL2 promotes vasculogenic mimicry and tumour aggressiveness in hepatocellular carcinoma. J. Cell Mol. Med..

[58] Shao J., Lu J., Zhu W., Yu H., Jing X., Wang Y.-L., Wang X., Wang X.-J. (2019). Derepression of LOXL4 inhibits liver cancer growth by reactivating compromised p53. Cell Death Differ..

[59] Osawa T., Ohga N., Akiyama K., Hida Y., Kitayama K., Kawamoto T., Yamamoto K., Maishi N., Kondoh M., Onodera Y. (2013). Lysyl oxidase secreted by tumour endothelial cells promotes angiogenesis and metastasis. Br. J. Cancer.

[60] Reynaud C., Ferreras L., Di Mauro P., Kan C., Croset M., Bonnelye E., Pez F., Thomas C., Aimond G., Karnoub A.E. (2017). Lysyl oxidase is a strong determinant of tumor cell colonization in bone. Cancer Res..

[61] Hu L., Wang J., Wang Y., Wu L., Wu C., Mao B., Maruthi Prasad E., Wang Y., Chin Y.E. (2020). LOXL1 modulates the malignant progression of colorectal cancer by inhibiting the transcriptional activity of YAP. Cell Commun. Signal..

[62] Palmieri V., Lazaris A., Mayer T.Z., Petrillo S.K., Alamri H., Rada M., Jarrouj G., Park W.-Y., Gao Z.-H., McDonald P.P. (2020). Neutrophils expressing lysyl oxidase-like 4 protein are present in colorectal cancer liver metastases resistant to anti-angiogenic therapy. J. Pathol..

[63] Boufraqech M., Patel D., Nilubol N., Powers A., King T., Shell J., Lack J., Zhang L., Gara S.K., Gunda V. (2019). Lysyl oxidase is a key player in BRAF/MAPK pathway-driven thyroid cancer aggressiveness. Thyroid.

[64] Liu J., Ping W., Zu Y., Sun W. (2014). Correlations of lysyl oxidase with MMP2/MMP9 expression and its prognostic value in non-small cell lung cancer. Int. J. Clin. Exp. Pathol..

[65] Zeltz C., Pasko E., Cox T.R., Navab R., Tsao M.-S. (2019). LOXL1 is regulated by integrin α11 and promotes non-small cell lung cancer tumorigenicity. Cancers.

[66] Peng D.H., Ungewiss C., Tong P., Byers L.A., Wang J., Canales J.R., Villalobos P.A., Uraoka N., Mino B., Behrens C. (2017). ZEB1 induces LOXL2-mediated collagen stabilization and deposition in the extracellular matrix to drive lung cancer invasion and metastasis. Oncogene.

[67] Mahjour F., Dambal V., Shrestha N., Singh V., Noonan V., Kantarci A., Trackman P.C. (2019). Mechanism for oral tumor cell lysyl oxidase like-2 in cancer development: Synergy with PDGF-AB. Oncogenesis.

[68] Park P.-G., Jo S.J., Kim M.J., Kim H.J., Lee J.H., Park C.K., Kim H., Lee K.Y., Kim H., Park J.H. (2017). Role of LOXL2 in the epithelial-mesenchymal transition and colorectal cancer metastasis. Oncotarget.

[69] El-Haibi C.P., Bell G.W., Zhang J., Collmann A.Y., Wood D., Scherber C.M., Csizmadia E., Mariani O., Zhu C., Campagne A. (2012). Critical role for lysyl oxidase in mesenchymal stem cell-driven breast cancer malignancy. Proc. Natl. Acad. Sci. USA.

[70] Erler J.T., Bennewith K.L., Cox T.R., Lang G., Bird D., Koong A., Le Q.-T., Giaccia A.J. (2009). Hypoxia-induced lysyl oxidase is a critical mediator of bone marrow cell recruitment to form the premetastatic niche. Cancer Cell.

[71] Chan N., Willis A., Kornhauser N., Ward M.M., Lee S.B., Nackos E., Seo B.R., Chuang E., Cigler T., Moore A. (2017). Influencing the tumor microenvironment: A phase II study of copper depletion using tetrathiomolybdate in patients with breast cancer at high risk for recurrence and in preclinical models of lung metastases. Clin. Cancer Res..

[72] Wang C., Xu S., Tian Y., Ju A., Hou Q., Liu J., Fu Y., Luo Y. (2019). Lysyl oxidase-like protein 2 promotes tumor lymphangiogenesis and lymph node metastasis in breast cancer. Neoplasia.

[73] Wu S., Zheng Q., Xing X., Dong Y., Wang Y., You Y., Chen R., Hu C., Chen J., Gao D. (2018). Matrix stiffness-upregulated LOXL2 promotes fibronectin production, MMP9 and CXCL12 expression and BMDCs recruitment to assist pre-metastatic niche formation. J. Exp. Clin. Cancer Res..

[74] Kasashima H., Yashiro M., Kinoshita H., Fukuoka T., Morisaki T., Masuda G., Sakurai K., Kubo N., Ohira M., Hirakawa K. (2016). Lysyl oxidase is associated with the epithelial-mesenchymal transition of gastric cancer cells in hypoxia. Gastric Cancer.

[75] Li Q., Zhu C.-C., Ni B., Zhang Z.-Z., Jiang S.-H., Hu L.-P., Wang X., Zhang X.-X., Huang P.-Q., Yang Q. (2019). Lysyl oxidase promotes liver metastasis of gastric cancer via facilitating the reciprocal interactions between tumor cells and cancer associated fibroblasts. EBioMedicine.

[76] Peng C., Liu J., Yang G., Li Y. (2018). Lysyl oxidase activates cancer stromal cells and promotes gastric cancer progression: Quantum dot-based identification of biomarkers in cancer stromal cells. Int. J. Nanomed..

[77] Tian J., Sun H.-X., Li Y.-C., Jiang L., Zhang S.-L., Hao Q. (2019). LOXL 2 Promotes the Epithelial-Mesenchymal Transition and Malignant Progression Of Cervical Cancer. OncoTargets Ther..

[78] Tanaka N., Yamada S., Sonohara F., Suenaga M., Hayashi M., Takami H., Niwa Y., Hattori N., Iwata N., Kanda M. (2018). Clinical implications of lysyl oxidase-like protein 2 expression in pancreatic cancer. Sci. Rep..

[79] Fang Y., Chang H.-M., Cheng J.-C., Klausen C., Leung P.C.K., Yang X. (2016). Transforming growth factor-β1 increases lysyl oxidase expression by downregulating MIR29A in human granulosa lutein cells. Reproduction.

[80] Feres-Filho E.J., Choi Y.J., Han X., Takala T.E., Trackman P.C. (1995). Pre- and post-translational regulation of lysyl oxidase by transforming growth factor-beta 1 in osteoblastic MC3T3-E1 cells. J. Biol. Chem..

[81] Voloshenyuk T.G., Hart A.D., Khoutorova E., Gardner J.D. (2011). TNF-α increases cardiac fibroblast lysyl oxidase expression through TGF-β and PI3Kinase signaling pathways. Biochem. Biophys. Res. Commun..

[82] Voloshenyuk T.G., Landesman E.S., Khoutorova E., Hart A.D., Gardner J.D. (2011). Induction of cardiac fibroblast lysyl oxidase by TGF-β1 requires PI3K/Akt, Smad3, and MAPK signaling. Cytokine.

[83] Taylor M.A., Amin J.D., Kirschmann D.A., Schiemann W.P. (2011). Lysyl oxidase contributes to mechanotransduction-mediated regulation of transforming growth factor-β signaling in breast cancer cells. Neoplasia.

[84] Kim D.J., Lee D.C., Yang S.-J., Lee J.J., Bae E.M., Kim D.M., Min S.H., Kim S.J., Kang D.C., Sang B.C. (2008). Lysyl oxidase like 4, a novel target gene of TGF-beta1 signaling, can negatively regulate TGF-beta1-induced cell motility in PLC/PRF/5 hepatoma cells. Biochem. Biophys. Res. Commun..

[85] Bierie B., Moses H.L. (2006). TGF-beta and cancer. Cytokine Growth Factor Rev..

[86] Najafi M., Farhood B., Mortezaee K. (2019). Extracellular matrix (ECM) stiffness and degradation as cancer drivers. J. Cell Biochem..

[87] Yoshikawa Y., Takano O., Kato I., Takahashi Y., Shima F., Kataoka T. (2017). Ras inhibitors display an anti-metastatic effect by downregulation of lysyl oxidase through inhibition of the Ras-PI3K-Akt-HIF-1α pathway. Cancer Lett..

[88] Baker A.M., Bird D., Lang G., Cox T.R., Erler J.T. (2013). Lysyl oxidase enzymatic function increases stiffness to drive colorectal cancer progression through FAK. Oncogene.

[89] Baker A.-M., Cox T.R., Bird D., Lang G., Murray G.I., Sun X.-F., Southall S.M., Wilson J.R., Erler J.T. (2011). The role of lysyl oxidase in SRC-dependent proliferation and metastasis of colorectal cancer. J. Natl. Cancer Inst..

[90] Hase H., Jingushi K., Ueda Y., Kitae K., Egawa H., Ohshio I., Kawakami R., Kashiwagi Y., Tsukada Y., Kobayashi T. (2014). LOXL2 status correlates with tumor stage and regulates integrin levels to promote tumor progression in ccRCC. Mol. Cancer Res..

[91] Barker H.E., Bird D., Lang G., Erler J.T. (2013). Tumor-secreted LOXL2 activates fibroblasts through FAK signaling. Mol. Cancer Res..

[92] Maruhashi T., Kii I., Saito M., Kudo A. (2010). Interaction between periostin and BMP-1 promotes proteolytic activation of lysyl oxidase. J. Biol. Chem..

[93] Jiang C., Zhou Y., Huang Y., Wang Y., Wang W., Kuai X. (2019). Overexpression of ADAMTS-2 in tumor cells and stroma is predictive of poor clinical prognosis in gastric cancer. Hum. Pathol..

[94] Porter S., Scott S.D., Sassoon E.M., Williams M.R., Jones J.L., Girling A.C., Ball R.Y., Edwards D.R. (2004). Dysregulated expression of adamalysin-thrombospondin genes in human breast carcinoma. Clin. Cancer Res..

[95] Lin Y.-M., Lin C.-W., Lu J.-W., Yeh K.-T., Lin S.-H., Yang S.-F. (2020). Decreased cytoplasmic expression of ADAMTS14 is correlated with reduced survival rates in oral squamous cell carcinoma patients. Diagnostics.

[96] Cai L., Xiong X., Kong X., Xie J. (2017). The role of the lysyl oxidases in tissue repair and remodeling: A concise review. Tissue Eng. Regen. Med..

[97] Peinado H., Zhang H., Matei I.R., Costa-Silva B., Hoshino A., Rodrigues G., Psaila B., Kaplan R.N., Bromberg J.F., Kang Y. (2017). Pre-metastatic niches: Organ-specific homes for metastases. Nat. Rev. Cancer.

[98] Doglioni G., Parik S., Fendt S.-M. (2019). Interactions in the (pre)metastatic niche support metastasis formation. Front. Oncol..

[99] Cao C., Lin S., Zhi W., Lazare C., Meng Y., Wu P., Gao P., Wei J., Wu P. (2020). LOXL2 expression status is correlated with molecular characterizations of cervical carcinoma and associated with poor cancer survival via epithelial-mesenchymal transition (EMT) phenotype. Front. Oncol..

[100] Barker H.E., Chang J., Cox T.R., Lang G., Bird D., Nicolau M., Evans H.R., Gartland A., Erler J.T. (2011). LOXL2-mediated matrix remodeling in metastasis and mammary gland involution. Cancer Res..

[101] Sakai M., Kato H., Sano A., Tanaka N., Inose T., Kimura H., Sohda M., Nakajima M., Kuwano H. (2009). Expression of lysyl oxidase is correlated with lymph node metastasis and poor prognosis in esophageal squamous cell carcinoma. Ann. Surg. Oncol..

[102] Kalikawe R., Baba Y., Nomoto D., Okadome K., Miyake K., Eto K., Hiyoshi Y., Nagai Y., Iwatsuki M., Ishimoto T. (2019). Lysyl oxidase impacts disease outcomes and correlates with global DNA hypomethylation in esophageal cancer. Cancer Sci..

[103] Shieh T.-M., Ko S.-Y., Chang S.-S., Chang K.-W., Shih Y.-H., Liu C.-J. (2011). Lysyl oxidase-like 3 mRNA expression indicates poor survival from oral squamous cell carcinoma. J. Dent. Sci..

[104] Albinger-Hegyi A., Stoeckli S.J., Schmid S., Storz M., Iotzova G., Probst-Hensch N.M., Rehrauer H., Tinguely M., Moch H., Hegyi I. (2010). Lysyl oxidase expression is an independent marker of prognosis and a predictor of lymph node metastasis in oral and oropharyngeal squamous cell carcinoma (OSCC). Int. J. Cancer.

[105] Zhan P., Shen X.-K., Qian Q., Zhu J.-P., Zhang Y., Xie H.-Y., Xu C.-H., Hao K.-K., Hu W., Xia N. (2012). Down-regulation of lysyl oxidase-like 2 (LOXL2) is associated with disease progression in lung adenocarcinomas. Med. Oncol..

[106] Ye M., Zhou J., Gao Y., Pan S., Zhu X. (2020). The prognostic value of the lysyl oxidase family in ovarian cancer. J. Clin. Lab. Anal..

[107] Choi J., Chung T., Rhee H., Kim Y.-J., Jeon Y., Yoo J.E., Noh S., Han D.H., Park Y.N. (2019). Increased expression of the matrix-modifying enzyme lysyl oxidase-like 2 in aggressive hepatocellular carcinoma with poor prognosis. Gut Liver.

[108] Umezaki N., Nakagawa S., Yamashita Y.-I., Kitano Y., Arima K., Miyata T., Hiyoshi Y., Okabe H., Nitta H., Hayashi H. (2019). Lysyl oxidase induces epithelial-mesenchymal transition and predicts intrahepatic metastasis of hepatocellular carcinoma. Cancer Sci..

[109] Han Y.-L., Chen L., Qin R., Wang G.-Q., Lin X.-H., Dai G.-H. (2019). Lysyl oxidase and hypoxia-inducible factor 1α: Biomarkers of gastric cancer. World J. Gastroenterol..

[110] Ma W., Li T., Wu S., Li J., Wang X., Li H. (2019). LOX and ACSL5 as potential relapse markers for pancreatic cancer patients. Cancer Biol. Ther..

[111] Lin S., Zheng L., Lu Y., Xia Q., Zhou P., Liu Z. (2020). Comprehensive analysis on the expression levels and prognostic values of LOX family genes in kidney renal clear cell carcinoma. Cancer Med..

[112] Celià-Terrassa T., Kang Y. (2018). Metastatic niche functions and therapeutic opportunities. Nat. Cell Biol..

[113] Piersma B., Hayward M.K., Weaver V.M. (2020). Fibrosis and cancer: A strained relationship. Biochim. Biophys. Acta Rev. Cancer.

[114] McDowell S.A.C., Quail D.F. (2019). Immunological regulation of vascular inflammation during cancer metastasis. Front. Immunol..

[115] Petrova V., Annicchiarico-Petruzzelli M., Melino G., Amelio I. (2018). The hypoxic tumour microenvironment. Oncogenesis.

[116] Tanikawa T., Wilke C.M., Kryczek I., Chen G.Y., Kao J., Núñez G., Zou W. (2012). Interleukin-10 ablation promotes tumor development, growth, and metastasis. Cancer Res..

[117] Özdemir B.C., Pentcheva-Hoang T., Carstens J.L., Zheng X., Wu C.-C., Simpson T.R., Laklai H., Sugimoto H., Kahlert C., Novitskiy S.V. (2014). Depletion of carcinoma-associated fibroblasts and fibrosis induces immunosuppression and accelerates pancreas cancer with reduced survival. Cancer Cell.

[118] Rhim A.D., Oberstein P.E., Thomas D.H., Mirek E.T., Palermo C.F., Sastra S.A., Dekleva E.N., Saunders T., Becerra C.P., Tattersall I.W. (2014). Stromal elements act to restrain, rather than support, pancreatic ductal adenocarcinoma. Cancer Cell.

[119] Cox T.R., Erler J.T. (2016). Fibrosis and cancer: Partners in crime or opposing forces?. Trends Cancer.

[120] Carapuça E.F., Gemenetzidis E., Feig C., Bapiro T.E., Williams M.D., Wilson A.S., Delvecchio F.R., Arumugam P., Grose R.P., Lemoine N.R. (2016). Anti-stromal treatment together with chemotherapy targets multiple signalling pathways in pancreatic adenocarcinoma. J. Pathol..

[121] Vennin C., Rath N., Pajic M., Olson M.F., Timpson P. (2020). Targeting ROCK activity to disrupt and prime pancreatic cancer for chemotherapy. Small GTPases.

[122] Roma-Rodrigues C., Mendes R., Baptista P.V., Fernandes A.R. (2019). Targeting tumor microenvironment for cancer therapy. Int. J. Mol. Sci..

[123] Ludwig J.A., Weinstein J.N. (2005). Biomarkers in cancer staging, prognosis and treatment selection. Nat. Rev. Cancer.

[124] Almacellas-Rabaiget O., Monaco P., Huertas-Martinez J., García-Monclús S., Chicón-Bosch M., Maqueda-Marcos S., Fabra-Heredia I., Herrero-Martín D., Rello-Varona S., de Alava E. (2020). LOXL2 promotes oncogenic progression in alveolar rhabdomyosarcoma independently of its catalytic activity. Cancer Lett..

[125] Vered M., Shnaiderman-Shapiro A., Schiby G., Zlotogorski-Hurvitz A., Salo T., Yahalom R. (2019). Markers of the pre-metastatic niche “knock on the door” of metastasis-free cervical lymph nodes in patients with oral cancer. Acta Histochem..

[126] Zhan P., Lv X.-J., Ji Y.-N., Xie H., Yu L.-K. (2018). Increased lysyl oxidase-like 2 associates with a poor prognosis in non-small cell lung cancer. Clin. Respir. J..

[127] Peinado H., Moreno-Bueno G., Hardisson D., Pérez-Gómez E., Santos V., Mendiola M., de Diego J.I., Nistal M., Quintanilla M., Portillo F. (2008). Lysyl oxidase-like 2 as a new poor prognosis marker of squamous cell carcinomas. Cancer Res..

[128] Zheng W., Wang X., Chen Q., Fang K., Wang L., Chen F., Li X., Li Z., Wang J., Liu Y. (2016). Low extracellular lysyl oxidase expression is associated with poor prognosis in patients with prostate cancer. Oncol. Lett..

[129] Janyasupab M., Lee Y.-H., Zhang Y., Liu C.W., Cai J., Popa A., Samia A.C., Wang K.W., Xu J., Hu C.-C. (2015). Detection of lysyl oxidase-like 2 (LOXL2), a biomarker of metastasis from breast cancers using human blood samples. Recent Pat. Biomark..

[130] Chen L.-C., Tu S.-H., Huang C.-S., Chen C.-S., Ho C.-T., Lin H.-W., Lee C.-H., Chang H.-W., Chang C.-H., Wu C.-H. (2012). Human breast cancer cell metastasis is attenuated by lysyl oxidase inhibitors through down-regulation of focal adhesion kinase and the paxillin-signaling pathway. Breast Cancer Res. Treat..

[131] Park J.S., Lee J.-H., Lee Y.S., Kim J.K., Dong S.M., Yoon D.S. (2016). Emerging role of LOXL2 in the promotion of pancreas cancer metastasis. Oncotarget.

[132] Gong R., Lin W., Gao A., Liu Y., Li J., Sun M., Chen X., Han S., Men C., Sun Y. (2019). Forkhead box C1 promotes metastasis and invasion of non-small cell lung cancer by binding directly to the lysyl oxidase promoter. Cancer Sci..

[133] Palamakumbura A.H., Vora S.R., Nugent M.A., Kirsch K.H., Sonenshein G.E., Trackman P.C. (2009). Lysyl oxidase propeptide inhibits prostate cancer cell growth by mechanisms that target FGF-2-cell binding and signaling. Oncogene.

[134] Bais M.V., Nugent M.A., Stephens D.N., Sume S.S., Kirsch K.H., Sonenshein G.E., Trackman P.C. (2012). Recombinant lysyl oxidase propeptide protein inhibits growth and promotes apoptosis of pre-existing murine breast cancer xenografts. PLoS ONE.

[135] Vora S.R., Palamakumbura A.H., Mitsi M., Guo Y., Pischon N., Nugent M.A., Trackman P.C. (2010). Lysyl oxidase propeptide inhibits FGF-2-induced signaling and proliferation of osteoblasts. J. Biol. Chem..

[136] Min C., Zhao Y., Romagnoli M., Trackman P.C., Sonenshein G.E., Kirsch K.H. (2010). Lysyl oxidase propeptide sensitizes pancreatic and breast cancer cells to doxorubicin-induced apoptosis. J. Cell Biochem..

[137] Wu M., Min C., Wang X., Yu Z., Kirsch K.H., Trackman P.C., Sonenshein G.E. (2007). Repression of BCL2 by the tumor suppressor activity of the lysyl oxidase propeptide inhibits transformed phenotype of lung and pancreatic cancer cells. Cancer Res..

[138] Min C., Kirsch K.H., Zhao Y., Jeay S., Palamakumbura A.H., Trackman P.C., Sonenshein G.E. (2007). The tumor suppressor activity of the lysyl oxidase propeptide reverses the invasive phenotype of Her-2/neu-driven breast cancer. Cancer Res..

[139] Palamakumbura A.H., Jeay S., Guo Y., Pischon N., Sommer P., Sonenshein G.E., Trackman P.C. (2004). The propeptide domain of lysyl oxidase induces phenotypic reversion of ras-transformed cells. J. Biol. Chem..

[140] Rachman-Tzemah C., Zaffryar-Eilot S., Grossman M., Ribero D., Timaner M., Mäki J.M., Myllyharju J., Bertolini F., Hershkovitz D., Sagi I. (2017). Blocking surgically induced lysyl oxidase activity reduces the risk of lung metastases. Cell Rep..

[141] Straub J.M., New J., Hamilton C.D., Lominska C., Shnayder Y., Thomas S.M. (2015). Radiation-induced fibrosis: Mechanisms and implications for therapy. J. Cancer Res. Clin. Oncol..

[142] Ejaz A., Greenberger J.S., Rubin P.J. (2019). Understanding the mechanism of radiation induced fibrosis and therapy options. Pharmacol. Ther..

[143] Guo K., Chen J., Chen Z., Luo G., Yang S., Zhang M., Hong J., Zhang L., Chen C. (2020). Triptolide alleviates radiation-induced pulmonary fibrosis via inhibiting IKKβ stimulated LOX production. Biochem. Biophys. Res. Commun..

[144] Jing X., Yang F., Shao C., Wei K., Xie M., Shen H., Shu Y. (2019). Role of hypoxia in cancer therapy by regulating the tumor microenvironment. Mol. Cancer.

[145] Sakthivel K.M., Hariharan S. (2017). Regulatory players of DNA damage repair mechanisms: Role in cancer chemoresistance. Biomed. Pharmacother..

[146] Tavora B., Reynolds L.E., Batista S., Demircioglu F., Fernandez I., Lechertier T., Lees D.M., Wong P.-P., Alexopoulou A., Elia G. (2014). Endothelial-cell FAK targeting sensitizes tumours to DNA-damaging therapy. Nature.

[147] Tang S.S., Trackman P.C., Kagan H.M. (1983). Reaction of aortic lysyl oxidase with beta-aminopropionitrile. J. Biol. Chem..

[148] Keiser H.R., Sjoerdsma A. (1967). Studies on beta-aminopropionitrile in patients with scleroderma. Clin. Pharmacol. Ther..

[149] Hajdú I., Kardos J., Major B., Fabó G., Lőrincz Z., Cseh S., Dormán G. (2018). Inhibition of the LOX enzyme family members with old and new ligands. Selectivity analysis revisited. Bioorg. Med. Chem. Lett..

[150] Tang S.S., Simpson D.E., Kagan H.M. (1984). Beta-substituted ethylamine derivatives as suicide inhibitors of lysyl oxidase. J. Biol. Chem..

[151] Findlay A.D., Foot J.S., Buson A., Deodhar M., Jarnicki A.G., Hansbro P.M., Liu G., Schilter H., Turner C.I., Zhou W. (2019). Identification and optimization of mechanism-based fluoroallylamine inhibitors of lysyl oxidase-like 2. J. Med. Chem..

[152] Schilter H., Findlay A.D., Perryman L., Yow T.T., Moses J., Zahoor A., Turner C.I., Deodhar M., Foot J.S., Zhou W. (2019). The lysyl oxidase like 2/3 enzymatic inhibitor, PXS-5153A, reduces crosslinks and ameliorates fibrosis. J. Cell Mol. Med..

[153] Chang J., Lucas M.C., Leonte L.E., Garcia-Montolio M., Singh L.B., Findlay A.D., Deodhar M., Foot J.S., Jarolimek W., Timpson P. (2017). Pre-clinical evaluation of small molecule LOXL2 inhibitors in breast cancer. Oncotarget.

[154] Tang H., Leung L., Saturno G., Viros A., Smith D., Di Leva G., Morrison E., Niculescu-Duvaz D., Lopes F., Johnson L. (2017). Lysyl oxidase drives tumour progression by trapping EGF receptors at the cell surface. Nat. Commun..

[155] Leung L., Niculescu-Duvaz D., Smithen D., Lopes F., Callens C., McLeary R., Saturno G., Davies L., Aljarah M., Brown M. (2019). Anti-metastatic Inhibitors of lysyl oxidase (LOX): Design and structure-activity relationships. J. Med. Chem..

[156] Smithen D.A., Leung L.M.H., Challinor M., Lawrence R., Tang H., Niculescu-Duvaz D., Pearce S.P., Mcleary R., Lopes F., Aljarah M. (2020). 2-Aminomethylene-5-sulfonylthiazole Inhibitors of lysyl oxidase (LOX) and LOXL2 show significant efficacy in delaying tumor growth. J. Med. Chem..

[157] Jiang H., Torphy R.J., Steiger K., Hongo H., Ritchie A.J., Kriegsmann M., Horst D., Umetsu S.E., Joseph N.M., McGregor K. (2020). Pancreatic ductal adenocarcinoma progression is restrained by stromal matrix. J. Clin. Investig..

[158] Rodriguez H.M., Vaysberg M., Mikels A., McCauley S., Velayo A.C., Garcia C., Smith V. (2010). Modulation of lysyl oxidase-like 2 enzymatic activity by an allosteric antibody inhibitor. J. Biol. Chem..

[159] Barry-Hamilton V., Spangler R., Marshall D., McCauley S., Rodriguez H.M., Oyasu M., Mikels A., Vaysberg M., Ghermazien H., Wai C. (2010). Allosteric inhibition of lysyl oxidase-like-2 impedes the development of a pathologic microenvironment. Nat. Med..

[160] Benson A.B., Wainberg Z.A., Hecht J.R., Vyushkov D., Dong H., Bendell J., Kudrik F. (2017). A Phase II randomized, double-blind, placebo-controlled study of simtuzumab or placebo in combination with gemcitabine for the first-line treatment of pancreatic adenocarcinoma. Oncologist.

[161] Hecht J.R., Benson A.B., Vyushkov D., Yang Y., Bendell J., Verma U. (2017). A Phase II, randomized, double-blind, placebo-controlled study of simtuzumab in combination with FOLFIRI for the second-line treatment of metastatic KRAS mutant colorectal adenocarcinoma. Oncologist.

[162] Grossman M., Ben-Chetrit N., Zhuravlev A., Afik R., Bassat E., Solomonov I., Yarden Y., Sagi I. (2016). Tumor cell invasion can be blocked by modulators of collagen fibril alignment that control assembly of the extracellular matrix. Cancer Res..

[163] Klepfish M., Gross T., Vugman M., Afratis N.A., Havusha-Laufer S., Brazowski E., Solomonov I., Varol C., Sagi I. (2020). LOXL2 inhibition paves the way for macrophage-mediated collagen degradation in liver fibrosis. Front. Immunol..

[164] Talantikite M., Lécorché P., Beau F., Damour O., Becker-Pauly C., Ho W.-B., Dive V., Vadon-Le Goff S., Moali C. (2018). Inhibitors of BMP-1/tolloid-like proteinases: Efficacy, selectivity and cellular toxicity. FEBS Open Bio..

[165] Gomez-Puerto M.C., Iyengar P.V., García de Vinuesa A., Ten Dijke P., Sanchez-Duffhues G. (2019). Bone morphogenetic protein receptor signal transduction in human disease. J. Pathol..

[166] Ovet H., Oztay F. (2014). The copper chelator tetrathiomolybdate regressed bleomycin-induced pulmonary fibrosis in mice, by reducing lysyl oxidase expressions. Biol. Trace Elem. Res..

[167] Chopra V., Sangarappillai R.M., Romero-Canelón I., Jones A.M. (2020). Lysyl oxidase like-2 (LOXL2): An emerging oncology target. Adv. Therap..

[168] Mohankumar A., Renganathan B., Karunakaran C., Chidambaram S., Konerirajapuram Natarajan S. (2014). Peptides derived from the copper-binding region of lysyl oxidase exhibit antiangiogeneic properties by inhibiting enzyme activity: An in vitro study. J. Pept. Sci..

[169] Karginova O., Weekley C.M., Raoul A., Alsayed A., Wu T., Lee S.S.-Y., He C., Olopade O.I. (2019). Inhibition of copper transport induces apoptosis in triple-negative breast cancer cells and suppresses tumor angiogenesis. Mol. Cancer Ther..

[170] Wang J., Luo C., Shan C., You Q., Lu J., Elf S., Zhou Y., Wen Y., Vinkenborg J.L., Fan J. (2015). Inhibition of human copper trafficking by a small molecule significantly attenuates cancer cell proliferation. Nat. Chem..

[171] Bahrami A., Khazaei M., Hasanzadeh M., ShahidSales S., Joudi Mashhad M., Farazestanian M., Sadeghnia H.R., Rezayi M., Maftouh M., Hassanian S.M. (2018). Therapeutic potential of targeting PI3K/AKT pathway in treatment of colorectal cancer: Rational and progress. J. Cell Biochem..

[172] Bahrami A., Khazaei M., Shahidsales S., Hassanian S.M., Hasanzadeh M., Maftouh M., Ferns G.A., Avan A. (2018). The therapeutic potential of pi3k/akt/mtor inhibitors in breast cancer: Rational and progress. J. Cell Biochem..

[173] Lv P.-C., Jiang A.-Q., Zhang W.-M., Zhu H.-L. (2018). FAK inhibitors in cancer, a patent review. Expert Opin. Ther. Pat..

[174] Kannaiyan R., Mahadevan D. (2018). A comprehensive review of protein kinase inhibitors for cancer therapy. Expert Rev. Anticancer Ther.

[175] Zhang X., Wang Q., Wu J., Wang J., Shi Y., Liu M. (2018). Crystal structure of human lysyl oxidase-like 2 (hLOXL2) in a precursor state. Proc. Natl. Acad. Sci. USA.

